# miRNA Landscape in Pathogenesis and Treatment of Vogt–Koyanagi–Harada Disease

**DOI:** 10.3389/fcell.2021.658514

**Published:** 2021-05-10

**Authors:** Fabian Vega-Tapia, Mario Bustamante, Rodrigo A. Valenzuela, Cristhian A. Urzua, Loreto Cuitino

**Affiliations:** ^1^Laboratory of Ocular and Systemic Autoimmune Diseases, Faculty of Medicine, Universidad de Chile, Santiago, Chile; ^2^Núcleo de Ciencias Biológicas, Facultad de Estudios Interdisciplinarios, Universidad Mayor, Santiago, Chile; ^3^Department de Health Science, Universidad de Aysén, Coyhaique, Chile; ^4^Department of Chemical and Biological Sciences, Faculty of Health, Universidad Bernardo O’Higgins, Santiago, Chile; ^5^Department of Ophthalmology, University of Chile, Santiago, Chile; ^6^Faculty of Medicine, Clínica Alemana Universidad del Desarrollo, Santiago, Chile; ^7^Servicio de Oftalmología, Hospital Clínico Universidad de Chile, Santiago, Chile

**Keywords:** miRNA, VKH, autoimmunity, inflammation, therapy

## Abstract

miRNAs, one of the members of the noncoding RNA family, are regulators of gene expression in inflammatory and autoimmune diseases. Changes in miRNA pool expression have been associated with differentiation of CD4^+^ T cells toward an inflammatory phenotype and with loss of self-tolerance in autoimmune diseases. Vogt–Koyanagi–Harada (VKH) disease is a chronic multisystemic pathology, affecting the uvea, inner ear, central nervous system, and skin. Several lines of evidence support an autoimmune etiology for VKH, with loss of tolerance against retinal pigmented epithelium-related self-antigens. This deleterious reaction is characterized by exacerbated inflammation, due to an aberrant T_*H*_1 and T_*H*_17 polarization and secretion of their proinflammatory hallmark cytokines interleukin 6 (IL-6), IL-17, interferon γ, and tumor necrosis factor α, and an impaired CD4^+^ CD25^*high*^ FoxP3^+^ regulatory T cell function. To restrain inflammation, VKH is pharmacologically treated with corticosteroids and immunosuppressive drugs as first and second line of therapy, respectively. Changes in the expression of miRNAs related to immunoregulatory pathways have been associated with VKH development, whereas some genetic variants of miRNAs have been found to be risk modifiers of VKH. Furthermore, the drugs commonly used in VKH treatment have great influence on miRNA expression, including those miRNAs associated to VKH disease. This relationship between response to therapy and miRNA regulation suggests that these small noncoding molecules might be therapeutic targets for the development of more effective and specific pharmacological therapy for VKH. In this review, we discuss the latest evidence regarding regulation and alteration of miRNA associated with VKH disease and its treatment.

## Introduction

miRNAs are short noncoding RNAs (20–23 nucleotides) that finely tune gene expression (Starega-Roslan et al., 2011). The best-known mechanism of action for gene regulation by miRNAs is post-transcriptional regulation through RISC-dependent binding or degradation of the target mRNAs, but other new functions have been described (Wu et al., 2010; Ramchandran and Chaluvally-Raghavan, 2017). Recognition of targets by the miRNA is based on the pairing of a short fragment (6–8 bases), which allows a single miRNA to bind to and regulate the activity of multiple targets, pathways, and, thus, cell function. This feature of miRNA also affects the immune system, and their dysregulation can lead to immune-related disorders such as autoimmunity.

The pathogenic role of miRNA in autoimmune diseases stems from their capability to regulate the activity of major immune-related pathways and immune cell function. For instance, Toll-like receptors (TLRs) and their signaling pathways are regulated by miRNAs, suggesting that miRNA dysregulation could reshape response toward endogenous DAMPs and foster autoimmunity (Nahid et al., 2011; He et al., 2014). Moreover, aberrant miRNA expression disrupts regulatory T cell (Treg) and tolerogenic dendritic cell function and phenotype stability (Li et al., 2014; Wu et al., 2018; Dekkema et al., 2019; Lyszkiewicz et al., 2019; Zhang et al., 2019; Chen et al., 2020; Geng et al., 2020; Tang et al., 2020). Involvement of miRNA in autoimmune diseases is also supported by the correlation of miRNA levels and many disease biomarkers. Let-7f expression is reduced in active systemic lupus erythematosus (SLE) and negatively correlates with disease activity index and proteinuria (Geng et al., 2020), whereas circulating exosomal miR-21 and miR-146a correlate with anti-SSA and anti-dsDNA levels, respectively (Li et al., 2020). Several miRNAs correlate with anti-citrullinated peptide antibodies levels and disease activity score in patients with rheumatoid arthritis (RA) (De La Rosa et al., 2020). miRNA may promote autoimmunity through direct binding to TLR-7/8 (Kim et al., 2016; Hegewald et al., 2020); therefore, general overexpression of miRNA may facilitate self-tolerance failure through mechanisms other than regulation on gene expression. In summary, miRNAs contribute to autoimmunity through the disruption of immune-related pathways, impairment of regulatory cell phenotype, or mounting immune response through TLR engagement.

## Vogt–Koyanagi–Harada Disease

Vogt–Koyanagi–Harada (VKH) disease is a rare autoimmune disease with ocular and systemic compromise. Ocular manifestations are characterized mainly by severe bilateral granulomatous panuveitis, exudative retinal detachments, and optic nerve edema, with eventual development of ocular pigmentary changes as late features in advanced phases of the disease. Systemic symptoms include tinnitus, hearing loss, vertigo, meningismus, vitiligo, and poliosis (O’keefe and Rao, 2017). A recent study suggests that delayed diagnosis and inadequate treatment lead to iris deterioration in patients with chronic recurrent disease (Chee and Win, 2021). VKH disease generally affects young women and is the leading cause of noninfectious uveitis with a known etiological factor in many high-risk populations, including India, Thailand, and Chile, and a major cause of panuveitis in Tunisia, Iran, Japan, and the Hispanic population in the United States (Liberman et al., 2015; O’keefe and Rao, 2017).

VKH etiopathogenesis is only partially understood; however, several studies provide evidence that the disease is caused by the immune reaction against pigmented cell–related autoantigens (Kobayashi et al., 1998; Otani et al., 2006). Therefore, understanding the mechanisms of immune dysregulation in the context of VKH is necessary to create more specific and effective therapies.

### CD4^+^ T Cells and Their Role in VKH Pathogenesis

As an autoimmune disorder, immune response against self is an underlying pathogenic mechanism of VKH, leading to the destruction and functional impairment of the retinal pigment epithelium and adjacent layers of the eye. Failure of tolerogenic mechanisms lead to the activation of self-reactive immune cells, including CD4^+^ T cells, which are major contributors to VKH. Increased CD4^+^ T cell population has been observed in the aqueous humor and cerebrospinal fluid of VKH patients (Norose et al., 1990, 1994; Ohta and Yoshimura, 1998). Initial phenotypical characterization of CD4^+^ T cells in VKH revealed an immune response shifted toward the T_*H*_1 subset with increased expression of activation markers CD25 and HLA-DR, the proinflammatory cytokine interferon γ (IFN-γ), and the transcription factor T-Bet (Norose and Yano, 1996; Li et al., 2005; Sugita et al., 2006). Notably, these T_*H*_1 cells have cytolytic activity on melanoma cells and cells expressing peptides related with pigmented tissues and express memory T cell markers, suggesting that loss of tolerance toward pigmented epithelium and long-term T_*H*_1 response are a crucial factor in VKH pathogenesis (Norose and Yano, 1996; Sugita et al., 2006). The introduction of the T_*H*_17 subset expanded the knowledge of the role of CD4^+^ T cells in autoimmunity. Interleukin 23 (IL-23) is a cytokine of the IL-12 family that induces differentiation of CD4^+^ T cells into the T_*H*_17 subpopulation to secrete the hallmark cytokine IL-17. IL-23 is increased in patients with active VKH (Wang et al., 2018) and is associated with active uveitis (Chi et al., 2008; Przepiera-Bedzak et al., 2016; Velez et al., 2016), and its administration enhances IFN-γ and IL-17 secretion in peripheral blood mononuclear cells (PBMCs) and isolated CD4^+^ T cells *in vitro* (Chi et al., 2007). Treatment-induced remission is associated with decreased expression of T_*H*_1 and T_*H*_17 cytokines and related transcription factors in PBMCs and CD4^+^ T cells (Liu et al., 2009).

Treg-inducing mechanisms seem to be defective in VKH; in a study published by Commodaro et al. (2010), no difference in the frequency of circulating Treg between controls and VKH patients, with or without active disease, was found. However, IL-10 and transforming growth factor β secretion was significantly stronger in the PBMCs from inactive VKH patients after *in vitro* stimulation, whereas IFN-γ was higher in active patients, without differences between control and inactive VKH groups (Commodaro et al., 2010). This study suggests that function, rather than number of Tregs, and maybe other regulatory cells, is impaired in VKH patients with active disease. In agreement with this, serum levels of the immunoregulatory cytokine IL-27 are decreased in patients with active VKH, which suppresses IL-17 expression and promotes IL-10 secretion in naive CD4^+^ T cells (Wang et al., 2012). Another immunoregulatory cytokine, IL-35, is also decreased in VKH patients, and culturing PBMCs with anti-CD3 and anti-CD28 antibodies in presence of IL-35 inhibits secretion of IFN-γ and IL-17 but enhances IL-10 release (Hu et al., 2019). Altogether, data show that deregulation of CD4^+^ T cells is an important event in VKH pathophysiology; therefore, understanding the mechanisms that cause these changes might be key for the development of effective therapies for this disease.

### Current Pharmacological Therapies for VKH

Systemic corticosteroids (CSs) (prednisolone) are the mainstay of clinical management of VKH, with evidence endorsing the use of high doses at early phases resulting in shorter treatment periods, reduced disease severity, and better subclinical manifestations (Chee et al., 2007; Jap et al., 2008; Kitaichi et al., 2008; Kawaguchi et al., 2010), whereas CS administration for at least 6 months is key to reduce the risk of recurrence (Lai et al., 2009; Errera et al., 2011). CS therapy is known to have several side effects, including systemic (diabetes, Cushing syndrome, osteoporosis) and eye-related features (cataract, glaucoma, visual impairment) (Valenzuela et al., 2020b).

Immunomodulatory therapy (IMT), including mycophenolate mofetil (MMF), methotrexate (MTX), cyclosporin A (CsA), and azathioprine, is usually used as a CS-sparing treatment with successful visual acuity improvement and reduction of sunset glow fundus development in some reports (Agarwal et al., 2006; Shen et al., 2016; Abu El-Asrar et al., 2017; Yang et al., 2018; Ei Ei Lin et al., 2020). Early use of CS and IMT combined as a first-line therapy increases the chances of remission and lowers the risk of chronic disease compared with CS monotherapy (Herbort et al., 2017, 2019).

Biologics have been introduced in uveitis management, and guidelines recommend them upon systemic CS/IMT treatment failure (Rosenbaum et al., 2019; Valenzuela et al., 2020b). Studies have shown that the use of adalimumab [anti-tumor necrosis factor α (TNF-α) antibody] (Couto et al., 2018; Hiyama et al., 2021) and rituximab (anti-CD20 antibody) (Abu El-Asrar et al., 2020) improves visual acuity, alleviates inflammation, and allows for CS tapering. Case reports have shown favorable results for the use of infliximab (anti-TNF-α) (Wang and Gaudio, 2008; Zmuda et al., 2013) and intravitreal bevacizumab (anti-VEGF-A) (Wu et al., 2009; Park et al., 2011) as treatment of VKH.

## miRNAs and Their Role in VKH Etiology and Treatment

### miRNAs as Mediators of VKH Pathogenesis

miRNAs have been implicated in the development of VKH disease. Asakage and colleagues recently reported their results on differentially expressed miRNAs (DEmiRs) in the serum of patients with noninfectious uveitis, including VKH, using a microarray approach (Asakage et al., 2020). The results revealed a set of 188 DEmiRs in VKH patients when compared with healthy controls (HCs), of which 59 DEmiRs were unique to VKH when compared with sarcoidosis and Behçet’s disease (BD). The authors used several approaches such as unsupervised hierarchical analysis and principal component analysis to show that VKH is related with a distinctive miRNA expression profile compared with uveitis of different etiology. Differential expression and copy number variation (CNV) in several miRNAs between VKH and BD have been described, probably due to the difference in the nature of these immunological disorders (autoimmune/adaptive in VKH vs. autoinflammatory/innate in BD) (Qi et al., 2013; Zhou et al., 2014; Hou et al., 2016). This distinctive miRNA expression pattern suggests that a specific miRNA-mediated mechanism is central to VKH pathogenesis. A summary of the findings and a brief discussion on the role of miRNAs involved in VKH are provided in Table 1.

**TABLE 1:** miRNAs associated with VKH.

miRNA	Association with VKH	Immunoregulatory effect	References
miR-20a	Hypermethylated promoter and downregulated in CD4^+^ T cells	Inhibits T_*H*_17 differentiation. Attenuates TCR signaling and regulates cytokine expression in activated CD4^+^ T cells.	Qi et al., 2013
miR-23a	Increased gene copy number	Correlates with IL-6 expression in PBMCs. Regulates the expression of IL-17 and HO-1.	Wu et al., 2009
miR-146a	Increased gene copy number	Promotes Treg function. Inhibits T_*H*_17 differentiation.	Wu et al., 2009
miR-182	Association with C allele of the rs76481776 variant	Evidence indirectly suggests a protective effect in VKH.	Liu et al., 2016
miR-301a	Decreased gene copy number	Promotes T_*H*_17 and TNF-α expression.	Wu et al., 2009
let-7g-3p	Good predictor of VKH	Unknown.	Park et al., 2011

**miR-20a**: Patients with active VKH have lower expression of miR-20a-5p in CD4^+^ T cells compared to HC, which is associated with a hypermethylated miR-20a-5p promoter (Chang et al., 2018). Overexpression of miR-20a-5p indirectly decreases IL-17 expression in VKH CD4^+^ T cells through the regulation of oncostatin M and CCL1 expression (Chang et al., 2018). Accordingly, a comprehensive analysis based on literature-supported miRNA-mRNA interactions found that miR-20a may suppress T_*H*_17 differentiation through the targeting of several regulators (Honardoost et al., 2015). Moreover, miR-20a expression increases upon T cell activation and inhibits T cell receptor signaling, while also decreasing the expression of CD69, IL-2, IL-8, and, especially, IL-10 (Reddycherla et al., 2015). Altogether, evidence suggests that miR-20a participates in a negative feedback loop that modulates CD4^+^ T cell activation and polarization.

**miR-23a**: A high copy number (>2) of the miR-23a coding gene is linked to VKH. This CNV directly correlates with increased miR-23a expression in PBMCs from HC, whereas overexpression of miR-23a increases IL-6 production in human retinal pigment epithelial cells (Hou et al., 2016). Conversely, miR-23a has been shown to restrain IL-17-mediated response by inhibiting the nuclear factor κB pathway (Hu et al., 2017) and to facilitate the expression of the immunoregulatory enzyme HO-1 by targeting its inhibitor Bach-1 (Su et al., 2020). Thus, miR-23a might act as a balancing factor in inflammation with opposing proinflammatory and anti-inflammatory roles. It is possible that the effect of miR-23a depends on the tissue it is expressed or is modified by the inflammatory milieu.

**miR-146a**: High miR-146a encoding gene copy number has been linked to VKH disease (Hou et al., 2016). However, no association with several miR-146 single-nucleotide polymorphisms (SNPs) was found in a similar study performed by the same authors (Zhou et al., 2014). The C allele of one of these SNPs (rs2910164 C > G) impairs nuclear processing of the pri-miR-146a, leading to lower mature miR-146a expression in PBMCs (Jazdzewski et al., 2008; Zhou et al., 2014), indicating that it has a functional impact. The lack of association of SNPs with VKH and the increased number of miR-146a encoding gene copies in these patients suggest that an aberrant overexpression of mature miR-146a, not its down-regulation, may have a role in the disease. Evidence supports a tolerogenic effect of miR-146a by enhancing Treg function (Lu et al., 2010; Zhou et al., 2015) and impairing T_*H*_17 differentiation (Liu et al., 2016; Li et al., 2017). How a high CNV of miR-146a gene is linked to VKH is still unknown.

**miR-182**: The rs76481776 SNP, located in the MIR182 gene, is associated with a limited expression of mature miR-182 in CC in versus TT or CT genotypes (Saus et al., 2010). A significant association with VKH was found for the C allele but not the T allele of the rs76481776 SNP in a Han Chinese cohort (Yu et al., 2014). Accordingly, the authors also report that CD4^+^ T cells from HC with the CC genotype have a lower expression of mature miR-182 compared with cells from donors carrying at least one T allele (Yu et al., 2014). Given the association of the C allele with VKH, the evidence suggests that miR-182 has protective role.

**miR-301a**: A low copy number of the MIR301A gene is associated with VKH in the Han Chinese population (Hou et al., 2016). Literature shows that miR-301a promotes T_*H*_17 differentiation and TNF-α production by targeting SNIP1 and PIAS3 (Mycko et al., 2012; He et al., 2016). There seems to be a contradiction between the low copy number and the proinflammatory effects of miR-301a in the context of VKH, although the relationship between CNV and expression has not been evaluated. A low copy number could sustain enough miR-301a expression without the activation of compensating negative feedback mechanisms, promoting inflammation.

**Let-7g-3p**: In one study, the authors identified a predictive panel of 24 miRNA in VKH patients, with let-7g-3p being the best predictor (Asakage et al., 2020), suggesting a strong link with disease development. A decreased expression of circulating let-7g-3p was found in Graves disease patients in remission (Hiratsuka et al., 2016). However, most of the knowledge on the immunoregulatory effects of let-7g is related with let-7g-5p (Yang et al., 2020). The role of let-7g-3p in VKH is still unclear.

### miRNA in Therapeutic Response

The current pharmacologic treatment for VKH includes immunosuppressive and immunomodulatory drugs, which are known to modify the expression of some of the previously reported VKH-related miRNA, accordingly to *in vitro*, animal model, and human studies evidence.

**Corticosteroids:** The expression of miR-20a, or the cluster miR17-92 in which it is located, is down-regulated by CS in mice lung tissue and murine T-cell lymphoma cell line (Moschos et al., 2007; Molitoris et al., 2011). The same effect is seen in miR-301a expression after CS stimulation (Moschos et al., 2007). Intracellular level of miR-23a is also down-regulated by dexamethasone (Dex) in both human endothelial cells and C2C12 myotubes, although the mechanism seems to be different as the one for miR-20a and miR-301a (Hudson et al., 2014b; Kwok et al., 2017). The use of Dex reverts the up-regulation of miR-146 induced by TNF-α in human bronchial epithelial cells *in vitro* and also in serum of pediatric patients with Crohn disease (Heier et al., 2016; Lambert et al., 2018). Finally, Dex decreases the level of miR-182 in preadipocytes, allowing C/EBPα-driven adipocyte differentiation (Dong et al., 2020).

**MMF:** The active metabolite of MMF, mycophenolic acid, is known to up-regulate miR-146a in T cells from SLE patients after treatment, according to microarray-based analysis and reverse transcription–quantitative polymerase chain reaction analysis (Tang et al., 2015).

**MTX:** RA patients who exhibit clinical improvement have higher blood levels of miR-146a-5p and other miRNAs at 4 months after MTX treatment initiation, supporting a mechanistic link between miR-146a expression and therapeutic response to MTX (Singh et al., 2019).

**CsA:** CsA-induced gingivae growth in rats occurs together with an up-regulation of miR-23a (Yang et al., 2017). The same effects over miR-23a expression are seen in primary mouse hepatocytes treated with CsA *in vitro* (Van Den Hof et al., 2014).

**Adalimumab:** A significant down-regulation of miR-146a-5p was observed in PBMCs from psoriasis patients after adalimumab treatment, reaching levels compared to that of the HC group (Mensa et al., 2018). Moreover, adalimumab reduced the expression of miR-146a in THP-1 and endothelial cells *in vitro* (Prattichizzo et al., 2016).

It is important to remark that none of the aforementioned publications established a direct relationship between changes of the miRNA expression and the therapeutic actions of the VKH-related drugs. How these drugs modify the levels of miRNAs is still under investigation, although the mechanisms behind might include (i) impairment of miRNA maturation, as CS down-regulates Dicer, Drosha, and DGCR8/Pasha and also induces G3BP1, all of them key miRNA processing enzymes (Smith et al., 2010; Kwok et al., 2017; Clayton et al., 2018); (ii) increasing exocytosis of miRNAs, making them less available intracellularly (Hudson et al., 2014a,b); (iii) histone modification in the promoter of miRNA genes by action of MMF or its active metabolite (Tang et al., 2015; Yang et al., 2015); and (iv) miRNA gene expression by indirect mechanisms that include extracellular adenosine signaling after MTX treatment (Yang et al., 2021). Less known are the mechanisms for CsA and adalimumab, although both drugs modify the profile expression of a number of different miRNAs (Gooch et al., 2017; Wcislo-Dziadecka et al., 2018).

A different but relevant aspect in the pharmacological treatment of VKH is the refractoriness to CS treatment [CS resistance (CSR)]. Recently, our group published a systematic review about CSR, a crucial issue in the management of uveitides such as VKH, which can be broadly described as refractory uveal inflammation despite the administration of high dose of CS (Valenzuela et al., 2020a). The VKH-related miRNA miR-182 confers CSR inhibiting apoptosis in lymphoma cells (Yang et al., 2012) and prevents CS-induced atrophy of skeletal muscle by targeting FOXO3a (Hudson et al., 2014a). Most data on CSR-related miRNAs involve molecules with unknown relationship with VKH; a summary of these findings is provided next as it could guide future research (Table 2). Evidence shows that a shift in the relative expression of the GR isoforms α and β after CS treatment constitutes a marker for CS sensitivity (Urzua et al., 2017, 2019). In this regard, miR-130b overexpression was found to inhibit GRα expression and conferred CSR to multiple myeloma cells (Tessel et al., 2011). Similarly, transfection of miR-331-3p mimic promotes CS sensitivity in several transformed cell lines by inhibiting JKN phosphorylation (Lucafo et al., 2020). Other pro-CSR miRNAs are miR-21, miR-29a and miR-222; conversely, miR-15b, miR-16, and miR-128b promote CS sensitivity in cancer cells (Kotani et al., 2009; Rainer et al., 2009; Wang et al., 2011; Glantschnig et al., 2019; Xu et al., 2019). Evidence for both pro- and anti-CSR effects of miR-221 and miR-124 has been reported (Kotani et al., 2009; Lv et al., 2012; Kim et al., 2015; Liang et al., 2017; Xu et al., 2019). Although CSR is a slightly different concept in the context of cancer, it also involves impairment of pharmacological response to CS; hence, some of these miRNAs might be involved in CSR in VKH patients as well.

**TABLE 2:** miRNAs involved in response to pharmacological treatment in VKH and corticosteroid resistance.

Corticosteroid response		

miRNA	Regulation by corticosteroids	References
miR-17-92 cluster (miR-20a)	Downregulation	Moschos et al., 2007; Molitoris et al., 2011
miR-20a	Downregulation	Moschos et al., 2007
miR-23a	Inhibition of maturation	Hudson et al., 2014b; Kwok et al., 2017
miR-146a	Downregulation	Heier et al., 2016; Lambert et al., 2018
miR-182	Downregulation	Dong et al., 2020
miR-301a	Downregulation	Moschos et al., 2007

**Corticosteroid resistance**		

**miRNA**	**Contribution to CSR**	**References**

miR-15b-16	Prevent CSR	Rainer et al., 2009
miR-21	Promotes CSR	Wang et al., 2011
miR-29a	Promotes CSR	Glantschnig et al., 2019
miR-124	Controversial	Lv et al., 2012; Kim et al., 2015; Liang et al., 2017
miR-128b	Prevents CSR	Kotani et al., 2009
miR-130b	Promotes CSR	Tessel et al., 2011
miR-182	Promotes CSR	Yang et al., 2012; Hudson et al., 2014a
miR-221	Controversial	Kotani et al., 2009; Xu et al., 2019
miR-222	Promotes CSR	Xu et al., 2019
miR-331-3p	Promotes CSR	Lucafo et al., 2020

**IMT**		

**Drug**	**Effect**	**References**

Mycophenolate mofetil	Upregulates miR-146a in SLE CD4^+^ T cells	Tang et al., 2015
Methotrexate	Downregulates miR-146a-5p but MTX-responsive patients have increased levels comapred to non-MTX-responsive patients.	Singh et al., 2019
Cyclosporine A	Upregulates miR-23a and miR-182	Van Den Hof et al., 2014; Yang et al., 2017
Adalimumab	Decreases miR-146a-5p	Prattichizzo et al., 2016; Mensa et al., 2018

## Conclusion

VKH is a complex disease with incompletely understood etiopathogenesis that requires aggressive long-term CS treatment with a high risk of wasting side effects. Although scarce, evidence supports a role of miRNAs in the development of VKH disease, therapeutic response, and even therapy resistance. Future research in the subject must aim to not only find the association of miRNAs with VKH and the use of therapy but also determine potential targets and functional changes caused by the differential expression of these regulatory RNAs. Successful progress in this task will contribute to establishing new pharmacological targets and biomarkers for disease activity and therapy response.

## Author Contributions

FV wrote the manuscript. MB, FV, RV, CU, and LC read, discussed, and revised the manuscript. All authors listed have made a substantial, direct and intellectual contribution to the work, and approved it for publication.

## Conflict of Interest

The authors declare that the research was conducted in the absence of any commercial or financial relationships that could be construed as a potential conflict of interest.
